# Sleep‐wake characteristics in a mouse model of severe traumatic brain injury: Relation to posttraumatic epilepsy

**DOI:** 10.1002/epi4.12462

**Published:** 2021-01-15

**Authors:** Sai Sruthi Konduru, Eli P. Wallace, Jesse A. Pfammatter, Paulo V. Rodrigues, Mathew V. Jones, Rama K. Maganti

**Affiliations:** ^1^ Department of Neurology University of Wisconsin School of Medicine and Public Health Madison WI USA; ^2^ Department of Neuroscience University of Wisconsin School of Medicine and Public Health Madison WI USA; ^3^ Cellular and Molecular Pathology Graduate Program University of Wisconsin School of Medicine and Public Health Madison WI USA

**Keywords:** NREM delta power, posttraumatic epilepsy, sleep spindles, sleep‐wake disturbances, Traumatic brain injury

## Abstract

**Study objectives:**

Traumatic brain injury (TBI) results in sequelae that include posttraumatic epilepsy (PTE) and sleep‐wake disturbances. Here, we sought to determine whether sleep characteristics could predict development of PTE in a model of severe TBI.

**Methods:**

Following controlled cortical impact (CCI) or sham injury (craniotomy only), CD‐1 mice were implanted with epidural electroencephalography (EEG) and nuchal electromyography (EMG) electrodes. Acute (1st week) and chronic (months 1, 2, or 3) 1‐week‐long video‐EEG recordings were performed after the injury to examine epileptiform activity. High‐amplitude interictal events were extracted from EEG using an automated method. After scoring sleep‐wake patterns, sleep spindles and EEG delta power were derived from nonrapid eye movement (NREM) sleep epochs. Brain CTs (computerized tomography) were performed in sham and CCI cohorts to quantify the brain lesions. We then employed a no craniotomy (NC) control to perform 1‐week‐long EEG recordings at week 1 and month 1 after surgery.

**Results:**

Posttraumatic seizures were seen in the CCI group only, whereas interictal epileptiform activity was seen in CCI or sham. Sleep‐wake disruptions consisted of shorter wake or NREM bout lengths and shorter duration or lower power for spindles in CCI and sham. NREM EEG delta power increased in CCI and sham groups compared with NC though the CCI group with posttraumatic seizures had lower power at a chronic time point compared with those without. Follow‐up brain CTs showed a small lesion in the sham injury group suggesting a milder form of TBI that may account for their interictal activity and sleep changes.

**Significance:**

In our TBI model, tracking changes in NREM delta power distinguishes between CCI acutely and animals that will eventually develop PTE, but further work is necessary to identify sleep biomarkers of PTE. Employing NC controls together with sham controls should be considered in future TBI studies.

**Key Points**

- Posttraumatic seizures were seen after CCI only, whereas epileptiform activity other than seizures was seen with sham injury as well
- Sleep‐wake disruptions consisted of shorter wake or NREM bout lengths, shorter duration or lower power for spindles, and increased NREM EEG delta power were seen in CCI and sham groups compared with NC
- NREM delta power was lower in CCI animals that developed posttraumatic seizures at a chronic time point compared with those without
- CT imaging showed that the sham injury group also had small brain lesions that may account for our findings
- These studies are relevant to further research in TBI models, to develop a sleep biomarker for PTE. The work is also relevant to humans with TBI as monitoring sleep phenotypes may not only predict risk, but also help develop therapies to prevent posttraumatic epilepsy

## 1 INTRODUCTION

According to CDC, there are over 5 million people with a traumatic brain injury (TBI)–related disability in the United States^1^, ^2^. TBI can lead to a wide range of sequelae including posttraumatic epilepsy (PTE), sleep disorders, cognitive and motor deficits, and neurobehavioral complications such as mood disorders or posttraumatic stress disorder (PTSD)^3^. Epidemiological data show that PTE is seen in about 10%–20% of patients with TBI in the civilian population but as high as 50% of injured military personnel^4^, ^5^. The 30‐year cumulative incidence of epilepsy is 2.1% for mild, 4.2% for moderate, and 16.7% for severe TBI^6^. In human and animal models alike, posttraumatic seizures that develop in the first week are defined as acute posttraumatic seizures and those that develop later are defined as late posttraumatic seizures or PTE^7^, ^8^. In humans, latency to PTE can be months to years after injury ^7^, ^8^. While cerebral contusions, intracranial hemorrhage, penetrating injuries, older age, and prolonged coma after injury are risk factors of PTE, it is currently impossible to predict who will develop PTE after TBI^9^.

Sleep‐wake disturbances are quite common after TBI, but the clear link between TBI severity and the development of sleep‐wake disturbances is not well established where they can be seen even after mild or repetitive mild TBI^10^, ^11^. Sleep‐wake disturbances can begin immediately after TBI and persist long after sustaining the injury^12^, ^13^. In humans, there is a spectrum of sleep complaints among TBI patients ranging from insomnia to hypersomnia^10^ with polysomnographic studies showing sleep fragmentation, increased awakenings after sleep onset, reduced REM sleep^13^, reduced nonrapid eye movement (NREM) sleep delta power, and reduced latencies on multiple sleep latency tests ^14^. In animal models of severe TBI, sleep disruptions include increased NREM sleep or shorter sleep/wake bouts and reduced time in REM sleep acutely^15^ or chronically^16^. Sleep‐wake disturbances and PTE can occur in the same person. While there are several hypotheses, exact mechanisms of PTE are unknown^17^. It is also unknown whether PTE and sleep disturbances have a shared mechanism although there is evidence of both hippocampal and thalamic GABA_A_ receptor subunit changes^18^ that favor deficits in synaptic inhibition. Interestingly, sleep and epilepsy appear to have a synergistic relationship whereby epilepsy disrupts sleep and sleep disruptions are a common trigger for seizures.

Here, we hypothesized that severe TBI results in sleep‐wake disturbances early on and that one or more sleep characteristics may serve as a biomarker of PTE.

## 2 METHODS

### 2.1 Animals

Adult male CD‐1 mice (~4 months old) were obtained from the Jackson Laboratory (Bar Harbor, ME). Mice were singly housed in their recording chambers during EEG recording days and were group‐housed otherwise. Mice were housed under a 12:12‐h light:dark cycle at 29 ± 1°C with access to food and water ad libitum. All procedures involving the use of animals were approved by the University of Wisconsin IACUC in accordance with the US Department of Agriculture Animal Welfare Act and the National Institutes of Health Policy on Humane Care and Use of Laboratory Animals.

### 2.2 Induction of TBI and electrode implantation

Briefly, CCI was delivered (n = 40) to induce a moderate‐to‐severe TBI to the right temporo‐parietal cortex and underlying dorsal hippocampus. Under isoflurane anesthesia and in a stereotaxic frame, a 4‐ to 5‐mm craniectomy was performed, centered at a location 5 mm posterior from bregma and 3 mm lateral. The dura was left intact. Isoflurane anesthesia was maintained at 2% for several minutes prior to delivery of the impact. Utilizing a Precision Cortical Impactor (Hatteras instruments, NC) with a 3‐mm‐diameter tip, a moderate‐to‐severe impact (2 mm depth, 5 m/s impact velocity, and 100‐200 msec dwell time) was delivered. Following the CCI, epidural stainless steel screw electrodes (fillister machine screws 0.90 UNM × 0.1 inch; Antrin Miniature Specialties, Fallbrook, Ca) were placed in the right frontal (1.5 mm anterior and lateral to bregma) and left parietal area (1.5mm posterior and lateral to bregma), as well as a reference electrode over the cerebellum (~6mm posterior to Lambda and 2 mm lateral). Additionally, two stainless steel wires were placed in the nuchal muscles to measure EMG for sleep scoring. Electrodes were connected to a head cap, and the electrode‐head cap assembly was fixed to the skull with dental acrylic. Sham injury animals (n = 24) received anesthesia for a similar duration with a craniectomy but no CCI delivered, but EEG and EMG electrodes were affixed as in TBI mice. A third control group (n = 6), employed later, consisted of CD‐1 mice that simply had EEG/EMG electrodes under anesthesia without a craniectomy.

Animals were allowed to recover for 1‐2 days postinjury, with appropriate postoperative care, and then transferred to individual recording chambers. Sequential twenty‐four‐hour video‐EEG recordings were acquired with an Xltek amplifier (Natus, Madison, WI), sampled at 1024 Hz, and filtered between 1 and 70 Hz. During the recording, animals were housed under diurnal conditions (12 hours light and 12 hours dark), in individual Plexiglas chambers (10 inches tall and 6 inches in diameter). Continuous one‐week‐long recordings occurred during the first week after surgery (acute) and then again (chronic) at months 1, 2, and 3 for TBI or sham groups and at week 1 and month 1 for NC controls. The acquired data were converted to EDF format for review and analysis. Sleep and seizure scoring was performed by reviewers blinded to treatment.

### 2.3 Analysis of seizures and interictal spikes

**FIGURE 1:**
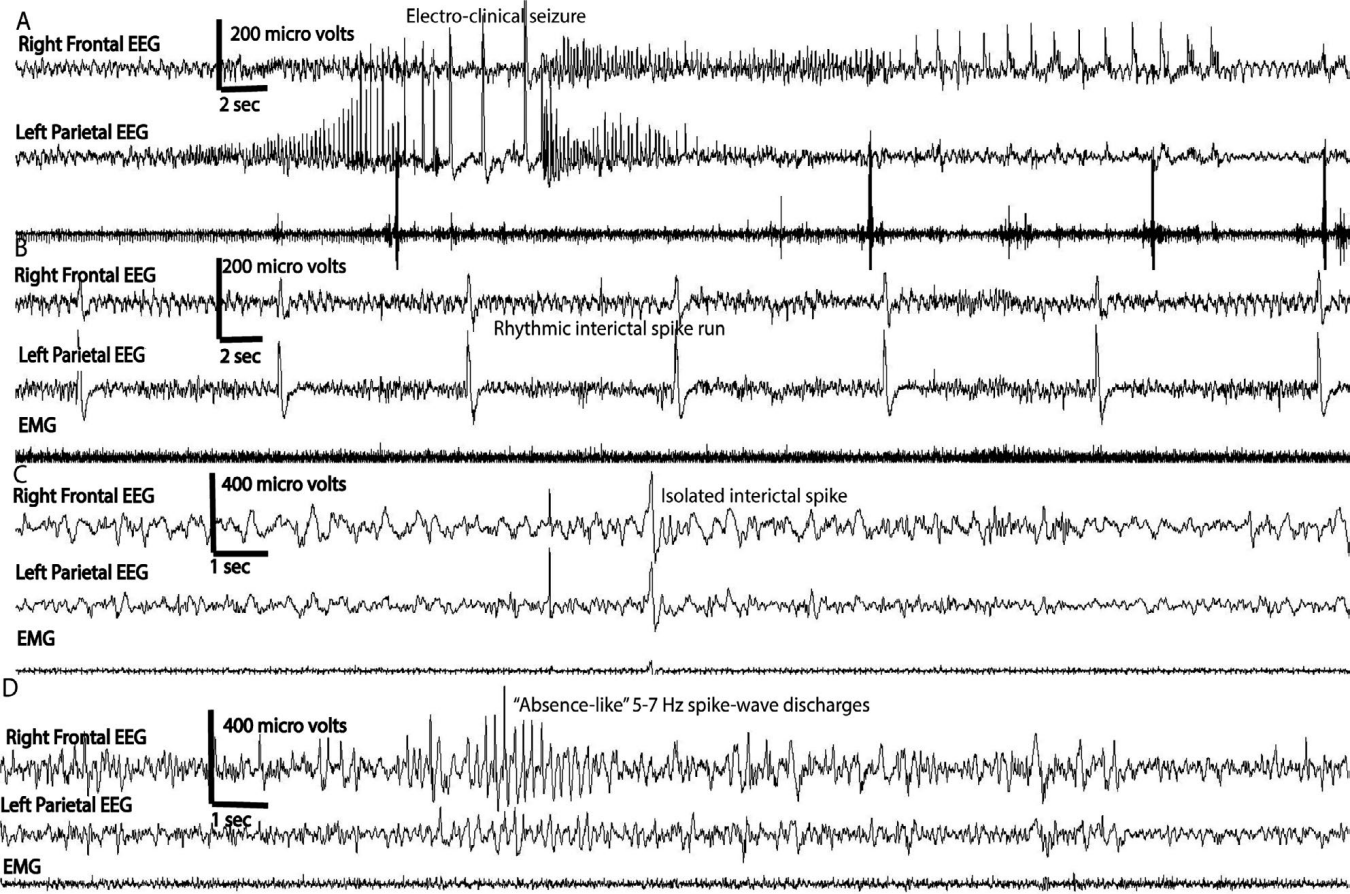
Epileptiform activity: Each animal had a right frontal and left parietal EEG leads, as well as an EMG electrode. Panel A shows EEG tracing from an animal having an electroclinical seizure in the first week of recording, where build‐up of rhythmic spike‐wave discharges is seen bilaterally in the frontal and parietal EEG. The behavioral correlate for this seizure consisted of behavioral arrest, head nodding, and hind limb stretching. In Panel B, a run of rhythmic or periodic interictal spikes is seen (60‐s window) in a mouse with CCI. Panel C shows an isolated interictal spike in a mouse with CCI. Panel D shows “absence‐like” spike‐wave discharges that is characterized by high‐amplitude spikes occurring at ~5‐7 Hz lasting about 4 s

**FIGURE 2:**
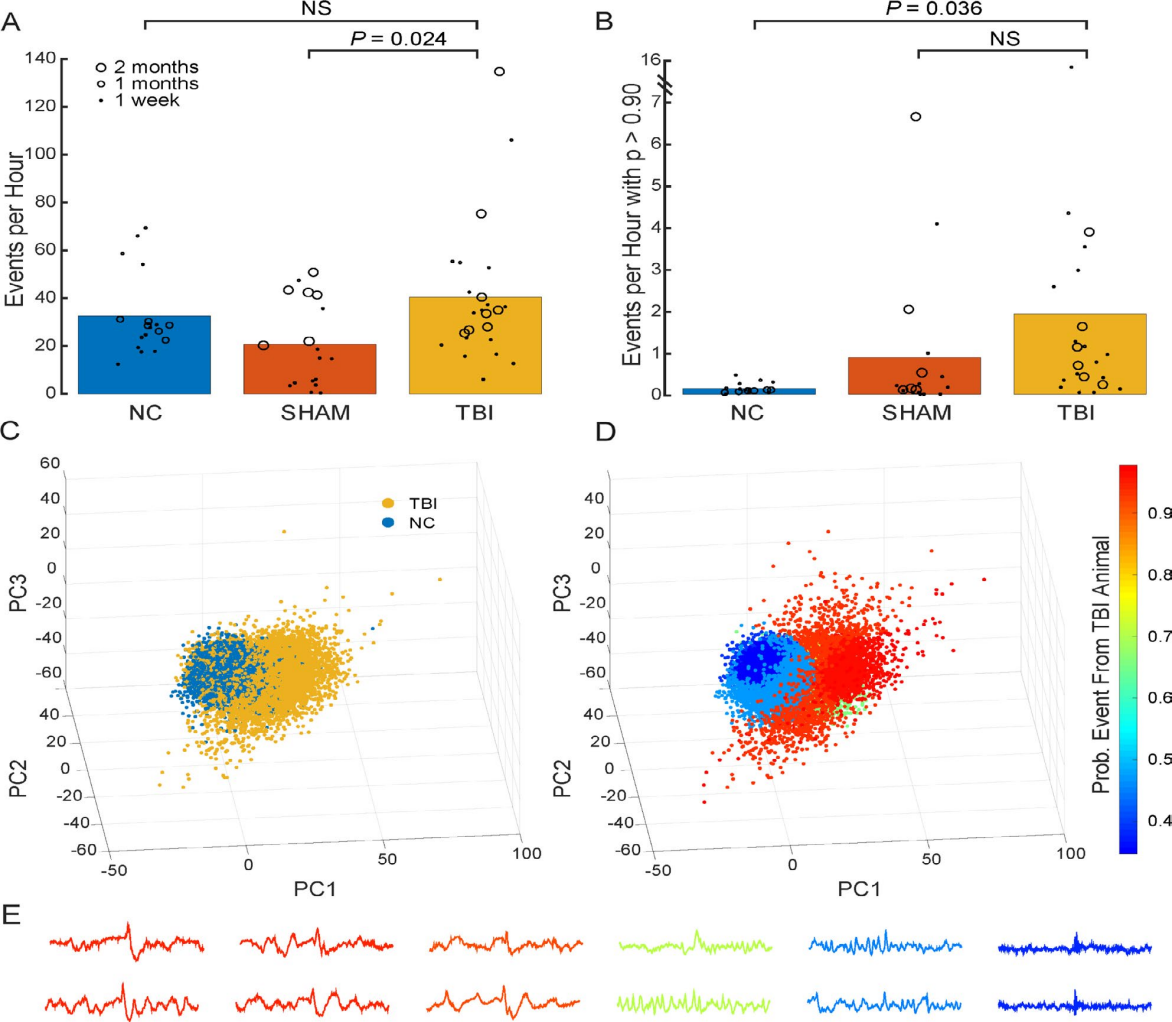
High‐amplitude event analysis: The frequency of high‐amplitude events detected from NC, sham, and TBI (CCI) EEG recordings, derived from the frontal EEG (3A). There are certain types of events identified using the algorithm in Pfammatter et al (2019) (those with a ratio greater than or equal to 0.90) that are predictive of TBI or TBI‐like injury (Panel B). Panels C‐D show how high‐amplitude events were projected into principal components space and how the event clusters were formed how TBI‐related ratios were applied to each cluster. Panel 3C shows the distribution of events from TBI and NC animals. The relative frequency of events from TBI animals to those from TBI and NC animals within each of nine clusters (Panel D) was used to identify events that have a high specificity (greater than 0.90 and those shown in Panel B) of being related to TBI treatment. Panel E shows a spectrum of events from those strongly related to TBI injury (left/red) to those less specifically related to the TBI treatment (right/green and blue)

All EEG data were notch‐filtered at 60 Hz using a Chebyshev type II digital filter and high‐pass (>0.5 Hz)‐filtered with a Chebyshev type I digital filter. For both filters, data were processed forward and backward to eliminate phase shift. All analyses presented in this manuscript used a right frontal EEG channel. EEG data were visually analyzed for all ictal and interictal activity. Interictal epileptiform activity including isolated spikes (defined as isolated spike and wave of < 200 milliseconds duration), spike trains or runs (defined as a rhythmic or semi rhythmic series of spikes lasting seconds or minutes), absence‐like spike‐wave discharges (defined as high‐amplitude (5‐7 Hz) events lasting 2‐4 seconds) was marked (Figure 1B‐D). The electrographic seizures (Figure 1A) were correlated with the behavioral characteristics on video and classified on a revised Racine scale^19^. We used an automated method ^20^, ^21^ for the detection of high‐amplitude interictal events from EEG records of the same files in which sleep data were analyzed. Briefly, we identified high‐amplitude events (as starting when signal crossed above five standard deviations from the mean and ending upon crossing back below one standard deviation). Events were considered as discrete spikes (as opposed to being part of a seizure) if their durations were briefer than 200 ms and they were separated from other events by more than 200 ms We then projected these events into principal components space and clustered events into nine groups (Figure 2E) using the first three principal components and a Gaussian mixture model. Within each cluster, we calculated the ratio of events belonging to animals from the TBI treatment to those from animals belonging to the CCI and NC treatments. This ratio (ranging from 0 to 1) provides the probability that any random event selected from within a cluster belongs to an animal that received CCI. From this analysis and for each EEG record, we calculated the number of high‐amplitude events per hour and the number of high‐amplitude events per hour with a ratio greater than or equal to 0.90 of being related to CCI (referred to as “CCI‐related” events) (Figure 2C,D). The significance of these latter events that are preferentially associated with TBI is that they may provisionally be considered as PTE‐related epileptiform events (or interictal spikes in those animals that experienced seizures).

### 2.4 Analysis of vigilance states, sleep spindle characteristics, and NREM delta power

**FIGURE 3:**
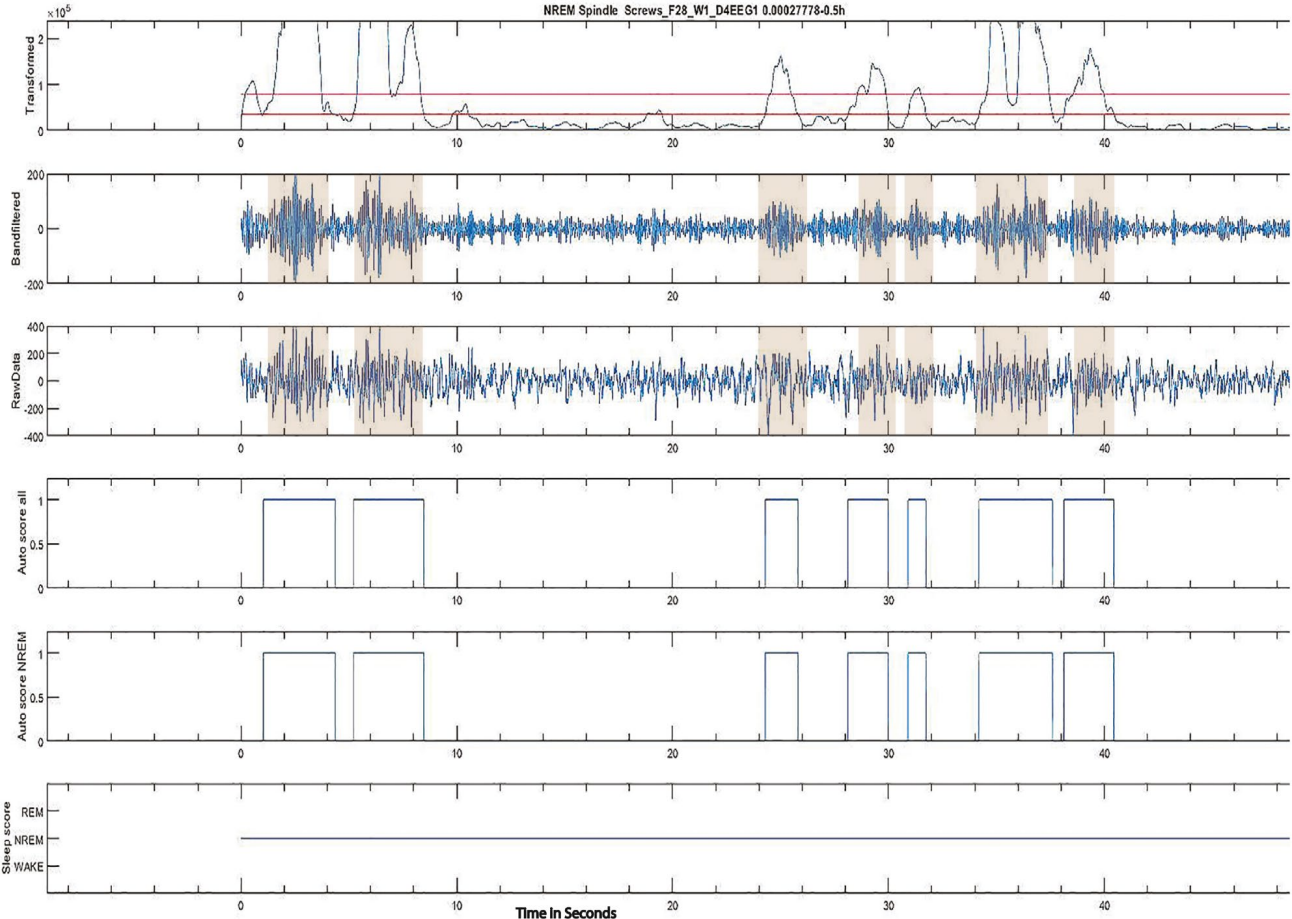
Spindle detection using a MATLAB algorithm: This figure depicts sleep spindle output detected in an NC control mouse on day 4 of the recording, through the automated MATLAB‐implemented algorithm. On the top row, cubed RMS signal of the frontal EEG, with detection thresholds, is seen. The 9‐ to 15‐hz band‐pass‐filtered signal is seen in row 2, and the raw EEG signal is seen in row 3 band‐pass‐filtered between 1 and 30 Hz. A spindle is detected when it crosses the amplitude thresholds (highlighted in shaded area for both algorithm detected and a corresponding raw EEG‐detected spindle). Rows 4 and 5 show the duration of each detected spindle by the algorithm. Bottom row shows the stage of sleep. Shaded area shows the spindles on raw EEG and 9‐ to 15‐hz band‐pass‐filtered EEG

EEG data were manually scored for vigilance states, in 4‐second epochs using Sirenia Sleep (Pinnacle Technologies, Lawrence, KS), divided into wake, NREM, or REM according to frontal EEG and EMG trace waveforms according to methods described previously^22^ (Figure S1). All epochs with seizures and artifactual EEG were excluded from sleep analysis. Day 4 and/or Day 5 of recording was selected randomly for sleep scoring. Manually scored sleep‐wake patterns were available for 6 days in NC controls (from 3 mice), 7 days in sham (from 7 mice), and 11 days in the TBI group (from 9 mice) at week 1; and for 6 days in NC (3 mice), 7 days for in (7 mice), and 12 days in the TBI group (12 mice) at month 1 or 2. Sleep parameters analyzed included total time spent in different vigilance states in 24 hours, bout number, and length for each vigilance state. Sleep spindles were analyzed using automated algorithm with a code that was provided by Uygun et al,^20^ in MATLAB (MathWorks, Natick, MA). This previously validated algorithm^23^ computes a root mean square (RMS) of the frontal EEG trace after band‐pass filtering at 9‐15 Hz. The RMS values are then cubed to facilitate placement of thresholds. A selection threshold of 1.5 × cubed RMS and detection threshold of 3.5 times cubed RMS were used to detect spindles. An example of the algorithm output in an NC control is shown in Figure 3. The output from the original algorithm yielded mean spindle density (number of spindles per minute of NREM sleep); mean spindle duration (sec); mean spindle peak amplitude (µV), and frequency (Hz) but was modified to yield spindle power (µV^2^). NREM EEG delta power was calculated for each scored 4‐second NREM epoch of frontal EEG by establishing power spectral density with fast Fourier transformation and integrating the power spectral density from 0.5 to 4 Hz. To account for interanimal variability, delta power values were normalized to the sum of power integrated over theta (5‐9 Hz), sigma (10‐14 Hz), and gamma (25‐100 Hz) bands.

### 2.5 Imaging

An imaging study was performed with computerized tomography (CT) scans in a subgroup of CCI and sham injury animals (n = 5 each). Formalin‐fixed mouse brains were CT‐scanned with a Siemens Inveon microCT (Siemens Medical Solutions USA, Inc, Knoxville, TN). All scans were acquired with the following parameters: 80 kVp, 1000 µA current, 700‐ms exposure time, 220 rotation steps with 600 projections, medium‐high magnification, and binning factor of 2. Raw data were reconstructed with filtered back‐projection applying the Shepp‐Logan filter using the high‐speed COBRA reconstruction software (Exxim Computing Corporation, Pleasanton, CA) yielding isotropic voxels of 31.5 microns. Analysis was conducted with Inveon Research Workplace (Siemens Medical Solutions USA, Inc, Knoxville, TN). We compared lesion size of any lesion identified in sham and CCI groups.

### 2.6 Data analysis

Time spent in different vigilance states and sleep efficiency across the 24 hours for NC, sham, and CCI was assessed using ANOVA with the post hoc Bonferroni‐Holm corrections. Then, sleep‐wake patterns or spindle density across the 24 hours was divided into different time bins (4‐hour bins for sleep‐wake and 6‐hour bins for spindles), and within‐group or between‐group differences were evaluated using a mixed‐model ANOVA, with treatment (ie, NC or sham or TBI) as a fixed‐effect variable and time as a random‐effect variable. Data for means and 95% confidence intervals (CI) were extracted. Differences in amount of time spent in each vigilance state at each individual time bin were compared using one‐way ANOVA with Bonferroni‐Holm post hoc correction. For each group included in ANOVA, data were not found to be skewed at a threshold less than those reported in [2]. NREM delta EEG power was analyzed using mixed‐model ANOVA at each time point [acute (week 1) or chronic (month 1 or 2)], with group (NC, sham, and CCI) as fixed‐effect, and time bin (ie, six 4‐hour bins) as a random‐effect variable. Multiple comparisons were completed with a Tukey‐Kramer post hoc correction. High‐amplitude event counts were compared between TBI/NC and TBI/sham treatment groups using two‐sample t‐tests with unequal variance. *P*‐values were corrected by the Bonferroni method where each *P*‐value was multiplied by the number of comparisons (two). Statistical analyses were conducted with an alpha value of 0.05.

## 3 RESULTS

### 3.1 Seizures following CCI

In acute or chronic video‐EEG recordings, electroclinical seizures were observed in about 23% (9 out of 40) of animals that had CCI, but none were observed in the sham or NC controls. Among the CCI group, 15 animals had both acute (week 1) and chronic (months 1, 2, and 3) recordings and two of these (~13%) had acute posttraumatic seizures. Chronic only (months 1, 2, and 3) recordings were available for 25 CCI animals, and of these, 7 (28%) developed PTE anywhere from months 1 to 3 after injury. The total number of Racine grade 3 to 5 seizures was relatively small with 10 seizures from 2 animals at week 1 (6 and 4 in each of the 2 mice), and 14 chronic posttraumatic seizures from 6 mice (range 1‐4 seizure in the 1‐week‐long recording). The behavioral correlate for the electrographic seizure consisted of behavioral arrest, head nodding, and hindlimb stretching that sometimes progressed to Racine class V seizures. The electrographic characteristics of the seizures consist of rhythmic fast activity that built into rhythmic spike‐wave discharges progressing bilaterally, similar to what was described previously by others (Figure 1A). Duration of the seizures varied from 20 to 160 seconds. No seizures were observed in sham or NC groups.

### 3.2 Epileptiform activity other than seizures

Visual inspection of EEG traces showed epileptiform activity not only in a larger proportion of CCI animals (15 out of 15 in acute recordings and 17 out of 25 in chronic recordings) but also in some sham group. The spike runs and absence‐like spike‐wave discharges were not quantified using any automated methods. Both these events lasted anywhere from 2 to 8 seconds in our sample and were not associated with any clear behavioral correlate such as freezing or wet dog shakes on video and were not counted as electroclinical seizures. The NC controls had no epileptiform activity on visual inspection. On automated analysis^20^ of high‐amplitude events, when all types of events are considered, no difference was found between the NC and CCI groups (t(37.31) = 0.525, uncorrected *P* = .603, corrected *P* > 1), but CCI had a greater number of such events compared with sham (t(35.55) = 2.63, uncorrected *P* = .012, corrected *P* = .024), (Figure 2A). However, when comparing high‐amplitude events whose waveforms were most predictive of whether the animal received CCI (those with a ratio of greater than 0.90^20^), we found that the CCI group had a greater number of these events compared with NC (t(21.07) = 2.58, uncorrected *P* = .018, corrected *P* = .036), but not compared with the sham group (t(34.48) = 1.29, uncorrected *P* = .205, corrected *P* = .409) (Figure 2B). Therefore, compared with NC, both CCI and sham displayed a preponderance of events indicative of having received CCI.

### 3.3 Vigilance state characteristics

**FIGURE 4:**
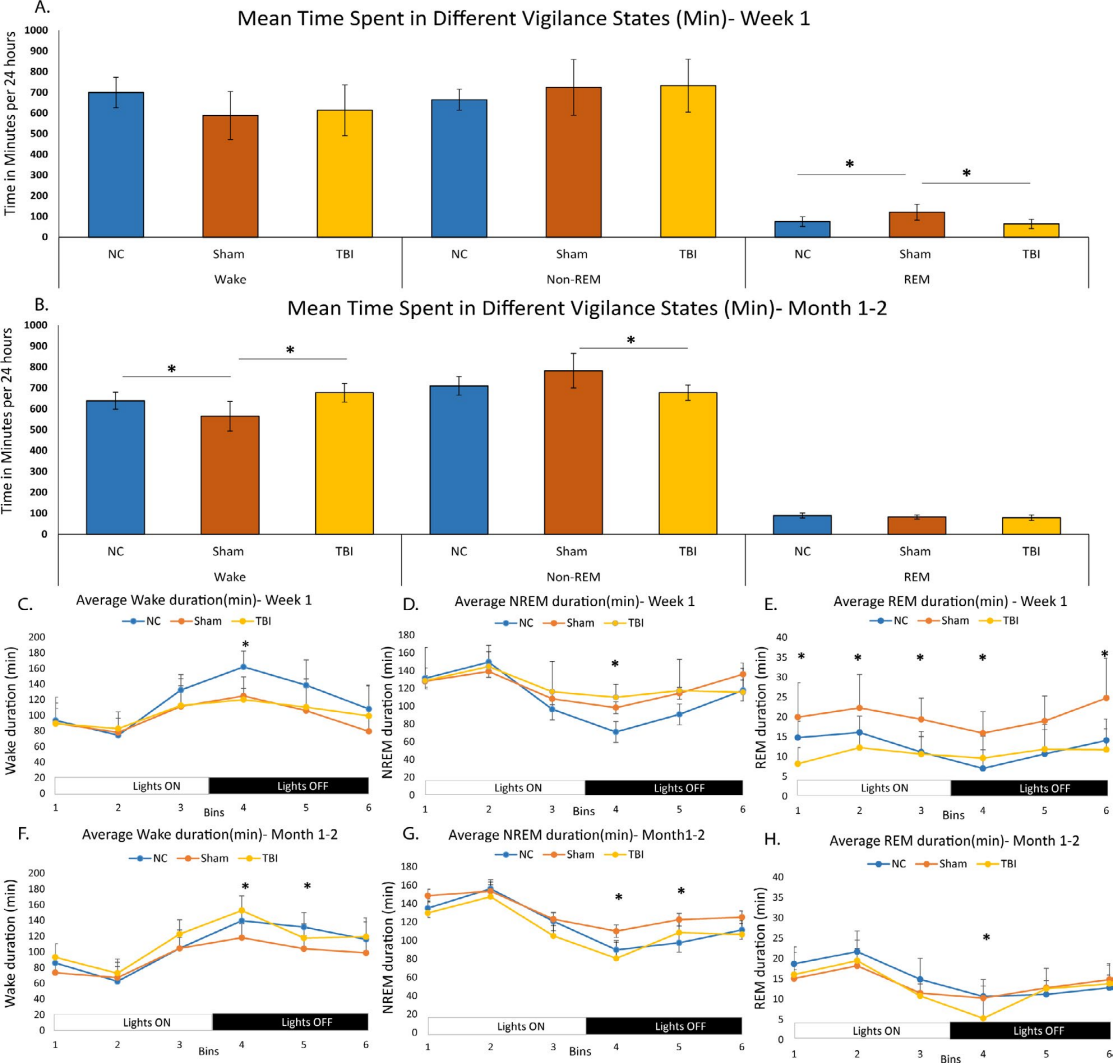
Vigilance state analysis: Figure 4 shows mean time spent in different vigilance states derived from frontal EEG and EMG signal, across 24 hours for week 1 (Panel A) and month 1 or 2 (Panel B). Panels C to E show oscillation of different vigilance states across 24 hours when examined in 4‐hour bins. Differences in average times spent in wake, NREM, and REM in each bin are shown in minutes at Week 1 (Panels C, D, and E) and at month 1 or 2 (Panels F, G, and H). Significance seen in any measure is indicated by an * in the figure. Acutely, NC (blue) had more time in awake in the 4th bin (6:30 PM to 10:30 PM), compared with sham (red) or CCI (orange) (wake: NC, 162 ± 8.23; sham, 124.74 ± 9.17; TBI, 120.02 ± 4.38, F(2, 23) = 10.03, *P* < .001*), and CCI had greater time in NREM (NC, 71.05 ± 6.55; sham, 98.41 ± 10.42; CCI, 110.01 ± 4.50, F(2, 23) = 7.694, *P* = .003*). Panel E shows time spent in REM (in minutes) at week 1 between the 3 groups (Bin 1: NC, 14.78 ± 1.69; sham, 19.93 ± 3.28; CCI, 8.17 ± 1.24, F(2, 23) = 9.073, *P* = .001*; Bin 2: NC, 16.09 ± 1.69; sham, 22.26 ± 3.18; CCI, 12.26 ± 1.11, F(2, 23) = 6.93, *P* = .004*; Bin 3: NC, 11.13 ± 1.60; sham, 19.39 ± 2.02; CCI, 10.62 ± 1.71, F(2, 23) = 6.72, *P* = .005*; Bin 4: NC, 7.00 ± 1.92; sham, 15.90 ± 2.05; CCI, 9.60 ± 1.66, F(2, 23) = 5.04, *P* = .01*; and Bin 6: NC, 14.08 ± 2.20; sham, 24.75 ± 3.79; CCI, 11.73 ± 1.59, F(2, 23) = 7.75, *P* = .003*) (Figure 4, E). Chronically, wake time was less for sham compared with CCI (in 4th bin) or NC (in 5th bin) (Bin 4: CCI, 152.68 ± 5.35 vs NC, 139.53 ± 4.44; sham, 117.90 ± 6.88, F(2, 24) = 9.25, *P* = .001*; and Bin 5: sham, 122.61 ± 6.15; NC, 97.45 ± 6.59; CCI, 108.54 ± 3.95, F(2, 24) = 4.61, *P* = .02). Sham had greater time spent in NREM sleep compared with CCI (4th bin) or NC (in 5th bin) (Bin 4: sham, 110.12 ± 6.99; NC, 89.86 ± 3.08; TBI, 80.57 ± 3.95, F(2, 24) = 7.50, *P* = .003*; and Bin 5: sham, 122.61 ± 6.15; NC, 97.45 ± 6.59; TBI, 108.54 ± 3.95, F(2, 24) = 4.61, *P* = .02*). Time spent in REM was less for TBI compared with both NC and sham in the 4th bin (TBI, 5.30 ± 1.33; NC, 10.68 ± 1.70; sham, 10.29 ± 1.10, F(2, 23) = 4.74, *P* = .01*)

The 24‐hour sleep efficiency was not different between the groups at week 1 (ANOVA: NC: 51.39 ± 5.11; sham: 58.92 ± 8.18; CCI: 52.60 ± 8.07; F(2, 21) = 1.688; *P* = .20). At month 1 or 2, however, ANOVA with post hoc Tukey test showed that sham group had greater sleep efficiency than CCI (NC: 55.59 ± 2.80; sham: 60.44 ± 5.30; CCI: 51.48 ± 2.33; F(2, 22) = 9.041; *P* = .001). At week 1, there were no differences in time spent in wake or NREM (in minutes) between NC vs sham injury vs CCI groups (wake: F(2, 21) = 1.78, *P* = .19; NREM: F(2, 21) = 0.7115, *P* = .50), but sham (121.18 ± 28.42) had greater time in REM compared with NC (75.28 ± 18.60, *P* < .05) or CCI (64.25 ± 13.20, *P* < .05) groups (Figure 4A) (ANOVA F(2, 21) = 9.16, *P* = .001). At month 1, however, there were differences in time spent in wake (F(2, 22) = 10.19, *P < *.001) and NREM (F(2, 22) = 8.29, *P* = .002) but not in REM (F(2, 22) = 1.49, *P* = .24). Post hoc comparisons showed that sham animals only had less time spent in wake (565.6 ± 52.51) compared with NC (638.35 ± 32.38, *P* < .05) or TBI (677.76 ± 20.66, *P* < .01) and greater time spent in NREM (782.98 ± 60.82) compared with TBI (677.72 ± 25.11, *P* < .01) (Figure 4B).

When vigilance states were divided into six 4‐hour bins across 24 hours, mixed‐model ANOVA showed a main effect of time where there was an oscillation seen in sleep‐wake pattern with greater sleep during lights on and greater wake during lights off (Figure 4‐E) (data shown for wake in Table S1). Comparison of time spent in different vigilance states in each of the 6 bins across 24 hours only showed difference in the 4th time bin (6:30 PM to 10:30 PM, first 4 hours of “lights off”) in week 1 where NC group spent more time awake and CCI group spent more time in NREM. The latter is indicative of early morning sleepiness in the CCI group (Figure 4C,D). The time spent in REM was much greater in sham compared with NC or CCI in multiple time bins (Figure 4C‐E). At month 1 or 2, result was mixed with sham animals having more NREM sleep and less wake in the 4th and 5th bins, which is 6:30 PM to 02:30am (Figure 4F,G), and CCI has less time spent in REM compared with both NC and sham in the 4th bin (Figure 4H).

Comparison of “bout number” of each vigilance state showed no consistent pattern. At week 1, sham groups had greater wake bout number than NC and greater NREM and REM bout number than NC or CCI (Figure S2A‐C). At month 1, CCI had greater number of wake bouts compared with either NC or sham (Figure S3D‐F). However, “bout length” (in seconds) analysis showed that sham and CCI had shorter wake, NREM, and REM bout length in both acute and chronic time points compared with NC (Figure S3A‐F). No difference was seen in bout lengths between sham and CCI groups. When the TBI group was subdivided into those with or without posttraumatic seizures, none of the above distinguished those with posttraumatic seizures.

### 3.4 NREM delta power analysis

**FIGURE 5:**
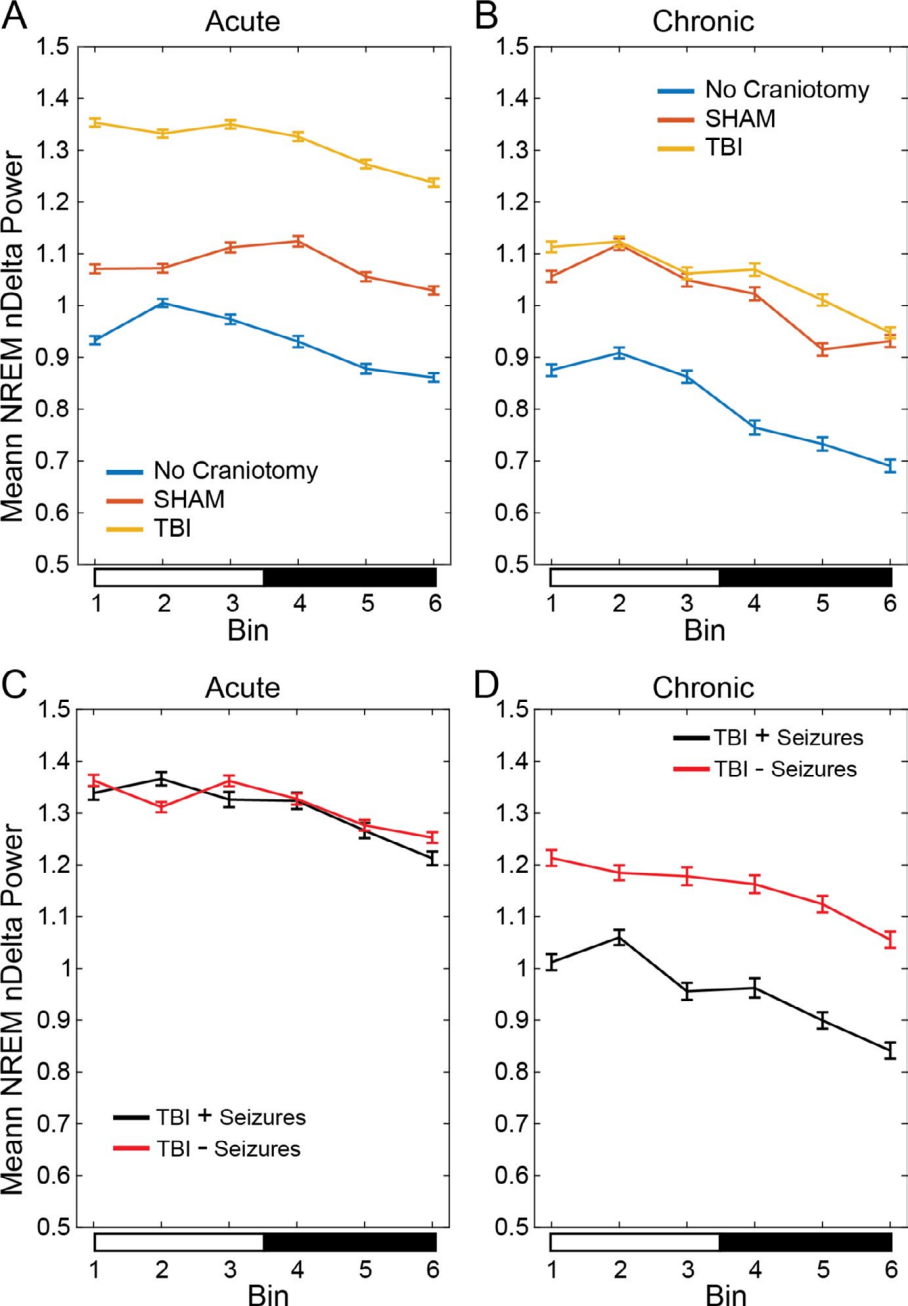
NREM delta power analysis: NREM delta power derived from the frontal EEG recording collected from animals that had sham, or CCI, or no craniotomy controls across 24 hours. Each recording was parsed into 4‐second epochs, which were manually scored for vigilance state (wake, NREM, or REM) and transformed into power spectral density plots. EEG power was calculated for delta (0.5‐4 Hz), theta (6‐9 Hz), sigma (10‐15 Hz), and gamma (25‐100 Hz) frequency bands. Delta power values were normalized to the sum power of delta + theta+sigma + gamma bands, and those normalized delta (nDelta) values associated with NREM sleep were isolated and further parsed into six 4‐hour bins for each day of recording. Mixed‐effect 2‐way ANOVAs with the Tukey‐Kramer‐corrected post hoc multiple comparison tests were run for recordings grouped by time relative to surgery, within 1 week of surgery (acute) or 1‐2 months following surgery (chronic). Binned NREM nDelta power of no craniotomy (NC, EEG only, blue) controls, sham (craniotomy + EEG, red), and CCI (craniotomy + CCI+EEG, yellow) were tested at acute (panel A) and chronic (panel B) time points and graphed as mean ± 95% confidence interval (95% CI). At the acute time point (panel A), analysis of NREM delta power showed a main effect based on time bin (F(5, 367 606) = 281.27, *P* < .05, Table S3B) and surgical group (F(2, 10) = 326.74, *P* < .05, Table S3C), though no significant interaction was seen (F(10, 367 606) = 0.57, *P* > .05). At the chronic time point (panel B), mixed‐effects ANOVA revealed a main effect of time (F(5, 201 367) = 571.31, *P* < .05, Table S3B) and surgical group (F(2, 10) = 168.49, *P* < .05, Table S3C), though no significant interaction was seen (F(10, 201 367) = 0.87, *P* > .05). To determine whether the presence of posttraumatic seizures differentially affected NREM nDelta power characteristics, we split those in the TBI group that exhibited seizures (TBI + seizures, black) and those that did not (TBI – seizures, red). Again, binned NREM nDelta power was tested with a mixed‐effects 2‐way ANOVA with Tukey‐Kramer‐corrected multiple comparisons, grouped by the presence or absence or seizures, at acute (panel C) and chronic (panel D) time points. At the acute time point (panel C), analysis of NREM delta power showed a significant effect of bin (F(5, 138 115) = 114.17, *P* < .05, Table S2E), but not the presence of seizure (F(1, 5) = 0.43, *P* > .05; Table S2F) or interaction (F(5, 138 115) = 0.68, *P* > .05). At the chronic time point (panel D), mixed‐effects ANOVA showed a significant effect of time bin (F(5, 75 405) = 153.07, *P* < .05; Table S2E), seizure presence (F(1, 5) = 146.07, *P* < .05; Table S2F), and interaction (F(5, 75 405) = 13.26, *P* < .05). Lighting condition is shown below each graph as lights on (open bar) and lights off (closed bar)

At week 1, the mean normalized EEG delta power was the lowest in NC, elevated in sham over NC, and highest in CCI animals (Figure 5A, Table S2A‐F, F(2, 10) = 326.7, *P* < .05). At the chronic time points, mean group NREM delta power of CCI animals decreased to levels similar to sham animals, while in both sham and CCI, it remained elevated over NC controls (Figure 5B, Table S2C, F(2, 10) = 168.49, *P* < .05). When group mean NREM delta power was compared between CCI animals that did or did not develop posttraumatic seizures, no difference was observed (Figure 5C, Table S2F, F(1, 5) = 0.43, *P* > .05). However, at the chronic time point, we found that CCI animals with detected seizures had lower NREM delta power than those CCI animals without detected seizures (Figure 5D, Table S2F, F(1, 5) = 146.07, *P* < .05). These findings suggest that acute increases in NREM delta power might be a useful diagnostic tool for TBI and its severity, as sham and CCI both exhibited elevated NREM delta power over NC controls. Acute NREM delta power may be a poor predictor of whether animals with severe TBI (CCI) would develop posttraumatic epilepsy, while NREM delta power weeks or months later may provide insights into the likelihood of developing PTE.

### 3.5 Sleep spindle analysis

**FIGURE 6:**
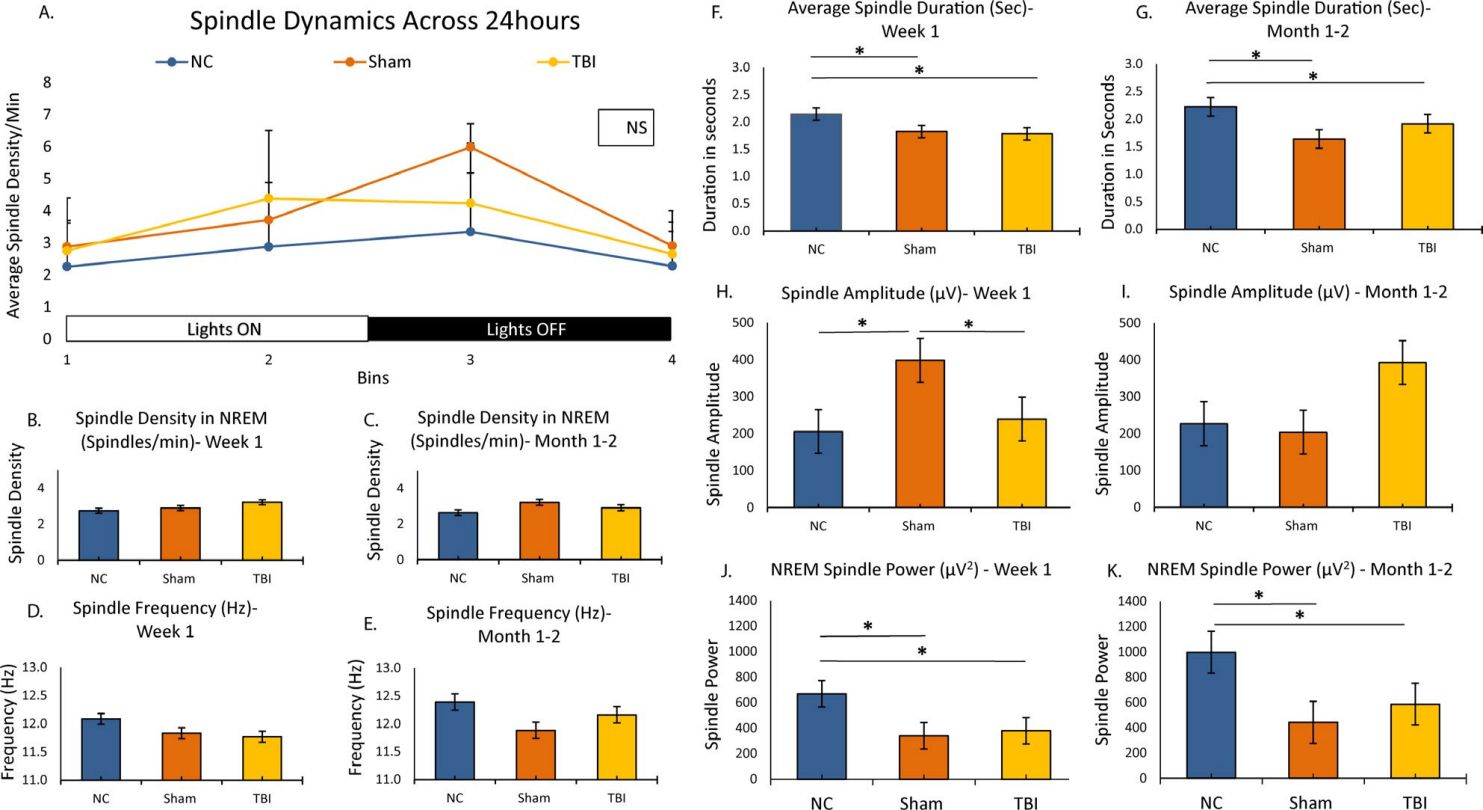
Sleep spindle analysis: Spindle characteristics are shown for NC, sham, and TBI animals at week 1 or month 1 or 2. Spindle dynamics are shown in panel A where mixed‐model ANOVA showed a main effect of time (F (2 21) = 8.02, *P* = .001) but not of treatment group (F (2, 21) = 1.45; *P* = .63). We also compared differences in spindle density between NC, sham, and TBI in each of the 4 bins using one‐way ANOVA, no differences were found (Bin 1, *P* = .60; Bin 2, *P* = .18; Bin 3, *P* = .13; Bin 4, *P* = .63). Differences in other spindle characteristics at week 1 and month 1 or 2 are then shown for: spindle duration (panels B and C); spindle frequency (panels D and E); spindle amplitude (panels F and G), spindle density (panels H and I); and spindle power (panels J and K). Any significant difference between groups is indicated by an *. ANOVA showed a main effect of group for spindle duration (in seconds) at week 1 (F(2, 28) = 5.58, *P* = .009) and at month 1 or 2 (F (2, 17) = 8.364, *P* = .003); spindle power at week 1 (F (2, 28) = 10.959, *P* < .001) and month 2 (F (2, 17) = 6.52, *P* < .001) and spindle amplitude at week 1 only (F (2, 28) = 5.30, *P* = .01). On post hoc comparisons, at week 1, spindle duration in seconds was lower in sham (1.83 ± 0.08, *P* < .05) or TBI (1.78 ± 0.09, *P* < .05) compared with NC (2.14 ± 0.08), but no difference was seen between sham and/or TBI. Similarly, at month 1 or 2, spindle duration was lower for sham (1.64 ± 0.07, *P* < .01) or TBI 1.92 ± 0.08 (*P* < .05) compared with NC (2.22 ± 0.11), but no difference was seen between sham and TBI (*P* = .15). For spindle power, post hoc comparison showed that NC (668.99 ± 69.05 µV^2^) had higher power than sham (339.21 ± 49.63 µV^2^, *P* < .01) or TBI (379.26 ± 33.38 µV^2^, *P* < .01) at week 1 and at month 1 or 2 also (NC, 996.92 ± 100.44 µV^2^; sham, 442.78 ± 115.99 µV^2^, *P* < .05; or TBI, 586.52 ± 88.33 µV^2^, *P* < .05), but there was no difference between sham and TBI either at week 1 or at month 1 or 2

First, we evaluated spindle dynamics across the 24 hours by examining the average spindle density per each 6‐hour bin of NREM sleep. Mixed‐model ANOVA showed a main effect of time where the spindles oscillate across the 24 hours, gradually increasing during lights on and gradually decreasing during lights off. However, there was no effect of treatment suggesting that no difference in spindle density was seen between the groups in any time bin (Figure 6A) (Table S3).

We then examined spindle duration, spindle frequency, amplitude, and power (data extracted for first 12 hours or lights on) both at week 1 and at month 1 or 2 of recordings among the 3 groups. Among these, spindle duration and spindle power were lower in CCI and sham compared with NC in acute or chronic recordings (Figure 6F,G,J,K, respectively). Spindle amplitude was also lower in CCI and sham compared with NC at week 1 (Figure 6H) but not at month 1 or 2 (Figure 6I). No differences were seen in other spindle characteristics between the groups (Figure 6B‐E) (Table S4). Spindle characteristics also did not differ in the CCI group with and without posttraumatic seizures, suggesting that spindle characteristics did not predict PTE in our sample.

### 3.6 Imaging data

The interictal epileptiform activity and sleep disruptions seen in both CCI and sham groups led us to perform imaging study (n = 5 each) to determine whether sham had a lesion as well (but also led us to employ NC controls later during this study). Voxel‐based analysis of the CT imaging showed a well‐defined lesion in the CCI group, but the sham group also had a lesion that is visually evident to the extent of the craniotomy on the images (Figure S4). However, the lesion volume was clearly larger for the CCI group compared with sham (CCI: 10.49 ± 7.2 mm^3^, sham: 0.82 ± 0.68 mm^3^; *P* = .039) (Figure S4) seen on the side of injury or craniotomy. What is evident from imaging is that the sham group likely had a milder form of brain injury that could have occurred during the craniotomy.

### 3.7 Correlations of high‐amplitude events with sleep‐wake patterns

We then examined whether interictal events contributed to sleep disruptions by correlating interictal high‐amplitude events per hour of recording with time spent in wake in each hour in the three groups. We found no correlation (Pearson's correlations: NC: *r* ^2^ = 0.25, *P* = .31; sham: *r* ^2^ = 0.07, *P* = .79; CCI: *r* ^2^ = 0.13, *P* = .60), suggesting that interictal high‐amplitude events themselves did not correlate with total wake time.

## 4 DISCUSSION

Overall, the key findings in our model of CCI are as follows: a) Following CCI, acute posttraumatic seizures were seen in 13% and late posttraumatic seizures (PTE) in 28% of animals, b) whereas no seizures were seen in sham and NC controls, epileptiform activity, high‐amplitude interictal events, and sleep‐wake disturbances were seen in both sham and CCI groups; c) sleep‐wake disturbances in acute and chronic recordings consisted of shorter wake and NREM sleep bout lengths, shorter duration, or lower power for sleep spindles in CCI and sham compared with NC; d) NREM delta power increased acutely in sham and CCI and lower for animals that developed posttraumatic seizures compared with those that did not at the chronic time point; e) sleep spindle duration and power were lower in CCI and sham than in NC; and f) finally, CT imaging showed that CCI animals had a large volume brain lesion, whereas sham injury animals had a small but definite lesion at their craniotomy site suggesting that they had at least a milder form of brain injury.

Our data are consistent with several prior studies in the CCI model. Seizures were reported anywhere from 9% to as much as 50% of animals following CCI^24^, ^25^, ^26^, ^27^ where they occur as early as within 24 hours of impact^25^, ^26^, ^27^ or late as 6‐9 months after injury^24^. Latency to spontaneous seizures was weeks to months in all the studies. We found acute posttraumatic seizures in the 1st week, whereas late posttraumatic seizures or PTE started anywhere from months 1 to 3 after injury. As our recordings were for 1 week at a time, we may have missed seizures during times when EEG was not recorded. We did not find any electroclinical seizures in our sham controls or NC controls. Others have reported “nonconvulsive seizures” and “absence‐like” events in Sprague Dawley rats^24^ that had CCI. We found isolated spikes, spike runs, or absence‐like spike‐wave discharges in the sham and CCI animals, similar to that of previous reports^24^, ^28^ where they lasted 2‐8 seconds without any clear behavioral correlate on video. Others reported the “absence‐like” spike‐wave discharges after sham treatment (or control) not only in Sprague Dawley rats^25^, ^29^, but also in outbred Sprague Dawley and Long Evans rats or even wild‐caught rats^30^. In a fluid percussion injury model, D'Ambrosio et al^31^ reported that control animals did not have the spike runs or absence‐like spike‐wave discharges until about 7‐8 months of age. In this model, the injury is given to the rostral parasagittal area, which can result in a “diffuse injury” whereas our model is a CCI model with focal injury. On automated analysis, we found high‐amplitude interictal events in all groups including NC similar to what we observed in saline‐treated C57BL6 mice in the Kainate model^20^. However, those with a unique morphology that was highly specific to the CCI group were also found in the sham but not in the NC group. Based on the data in the Kainate model, we expected a much higher index of high‐amplitude events in animals that eventually develop seizures, but we saw no difference in these events among CCI group with and without seizures.

Sleep‐wake disturbances were reported in rodent models of TBI including the CCI^32^, ^33^, ^34^, ^35^, ^36^, ^37^, lateral or midline fluid percussion injury^16^, ^38^, ^39^, ^40^, ^41^, ^42^, and weight drop^43^, ^44^, but the data are conflicting. In CCI models, increased NREM sleep, shorter wake bouts, increased sleep‐wake fragmentation ^32^, and increased latency to reach peak sleep^33^ and hypersomnia acutely^34^ were reported. In fluid percussion injury models, one study reported no changes in sleep‐wake patterns after TBI^41^ and yet another reported increased mean percent time spent sleeping in the first week but not 2‐5 weeks postinjury^40^. In a weight drop model of mild TBI, increased long wake bouts were reported acutely^44^. Our analyses were at an acute and chronic time points, but no consistent sleep‐wake disturbances were attributable solely to the CCI. We found the normal expected oscillation of sleep/wake across lights on and lights off, suggesting that the normal diurnal sleep‐wake pattern was preserved in all groups. The greater time in NREM in the 4th bin (6:30 PM to 10:30 PM, which is the first 4 hours of rodent wake time) in sham and CCI suggests that craniotomy itself resulted in “early morning sleepiness.” Furthermore, “sleep fragmentation” with shorter wake or NREM bouts were seen in CCI or sham injury groups acutely or chronically, suggesting that any disruptions were craniectomy‐related rather than due to CCI.

Sleep spindle characteristics have been described as a potential biomarker of PTE in previous studies, but data are conflicting. One study reported increased spindle frequency and duration in mice that had TBI compared with their sham controls^45^. Another group reported shorter duration of spindles during transition from NREM to REM sleep in rats with TBI compared with a sham group and even among TBI, those that developed seizures had shorter duration of spindles compared with those that did not^46^. The same group later reported that spindle characteristics could be potential predictors of PTE^47^. We demonstrated the normal dynamics of spindles or the normal 24‐hour oscillation of spindles across lights on and lights off^48^ and a relative decrease in spindle duration and power in sham and CCI groups compared with NC controls both acutely and chronically. No spindle characteristic was predictive of PTE in our model.

Our finding of changes in NREM delta power was unique. One prior study reported that NREM delta power increases transiently after CCI when examined sequentially from day 1 to 30 after injury in a CCI model^37^. The NREM delta power is a marker of homeostatic sleep pressure that normally oscillates across the 24 hours, where it declines with sleep (as the homeostatic sleep drive decreases) and increases during the wake period (as the homeostatic drive for sleep or sleep pressure increases)^49^. Prior studies in rodents including CD‐1 mice showed that NREM delta power decreases progressively during lights on when rodents sleep and increases during lights off as they spend more time awake^44^, ^50^, ^51^. In our model, interestingly, we did not find these dynamics or the oscillation acutely or chronically. More importantly, we found an increase in the mean NREM delta power that followed injury severity from NC to sham to CCI especially during the first week, but remained high chronically, in sham and CCI groups than NC control. Lastly, when we parsed CCI group into those with and without seizures, we noted a difference where CCI animals with seizures had mean NREM delta power that is similar to levels seen in NC controls at a chronic time point. Seizures or interictal spikes can result in increased synaptic strengthening and contribute to increased NREM delta power^52^. Thus, in the CCI model, the presence of epileptiform activity seen in both sham and CCI groups might have possibly led to the increased NREM delta power in both. However, contrary to conventional wisdom, we saw no difference between those that did or did not develop seizures. Future studies may resolve independent impact of seizures and interictal spikes on NREM delta power.

Given that our data showed interictal phenomena and sleep‐wake disruptions in CCI and sham groups alike, we decided to employ an NC control group but also followed with an imaging study in sham and CCI cohorts. Our imaging data showed that indeed animals with sham injury also had a visually evident lesion to the extent of the craniectomy, though on quantification, the lesion was substantially smaller in volume than in the CCI group. Taken together with the findings on sleep‐wake changes and interictal phenomena in both CCI and sham groups, we speculate that perhaps the craniectomy itself in sham animals resulted in some degree of cerebral injury that contributed to these findings. Thus, future studies should consider employing NC controls along with equal number of sham and CCI to better identify differences in various sleep characteristics or epileptiform activity.

There are several limitations to our interpretation of the data. First, our sample sizes are small. We may have missed seizures or even nonconvulsive events when animals were not being recorded. We have not quantified “absence‐like” discharges or spike runs. Sleep scoring in 4‐second epochs is extremely labor‐intensive, and data were analyzed in a proportion of animals. Analysis of sleep in all animals of all cohorts using automated methods is a future strategy. Future studies could also determine the chronology of changes in sleep or epileptiform activity after TBI through continuous chronic recordings in all groups of animals including NC controls with equal sample sizes. A unique future opportunity is to design studies to sequentially perform recordings before and after CCI, such that every animal becomes its own control allowing group and within animal differences. Our craniectomy itself resulted in some degree of cerebral injury in the sham group of animals, which is a weakness of the study. A histopathology study might have yielded definitive information of any degree of brain injury in the sham group. In the future, perhaps employing a no craniotomy control along with sham, may be more prudent. A methodological improvement during craniectomy in sham animals to avid cerebral injury should also be a consideration.

## 5 CONCLUSIONS

In conclusion, CCI results in acute posttraumatic seizures or PTE in a proportion of animals, while interictal spike runs or “absence‐like” spike‐wave discharges were seen in both CCI and sham injury groups. Furthermore, high‐amplitude interictal events were seen in all groups, though the ones with high specificity for TBI were seen to a far greater extent in those with CCI compared with NC. Similarly, any sleep‐wake disruptions were seen in CCI and sham groups. The significance of decline in NREM delta power in animals that developed PTE at the chronic time point is unclear, but further work is needed to understand how NREM delta power evolves after TBI and its relation to development of PTE. Given the changes we observed in interictal phenomena, sleep‐wake patterns, spindles, and NREM delta power, as well as imaging data in sham injury animals, using animals with no craniotomy of equal number to sham injury and CCI is a strong consideration for future studies. These studies are relevant to further research in TBI models, to develop a sleep biomarker for PTE, and may develop therapies to prevent posttraumatic epilepsy.

## CONFLICT OF INTEREST

None of the authors have any conflicts of interests to disclose. We confirm that we have read the Journal's position on issues involved in ethical publication and affirm that this report is consistent with those guidelines.

## Supporting information

Supplementary Material

## Data Availability

A previous version of this manuscript had been posted at BioRxiv at https://www.biorxiv.org/content/10.1101/2020.06.16.137034v1. The current version published at Epilepsy Open is a revised version following the journal's peer review process.
