# Supplementary material for: Sleep‐wake characteristics in a mouse model of severe traumatic brain injury: Relation to posttraumatic epilepsy
Source: Epilepsia Open. 2021 Jan 15;6(1):181–94. doi: 10.1002/epi4.12462 (PMC7918302; doi:10.1002/epi4.12462)
Supplement: Supplementary file 1 — Supplementary Material [file EPI4-6-181-s001.docx]

Supplementary Data:

Title: **Sleep-wake Characteristics in a Mouse Model of Severe Traumatic Brain Injury: Relation to Post-Traumatic Epilepsy**.

Sai Sruthi Konduru^1^, Eli P Wallace^1, 2, 3^, Jesse A Pfammatter^2^, Paulo V Rodrigues^1^, Mathew V Jones^2^, Rama K Maganti^1^

1. Department of Neurology, University of Wisconsin School of Medicine and Public Health, Madison WI
2. Department of Neuroscience, University of Wisconsin School of Medicine and Public Health, Madison WI
3. Cellular and Molecular Pathology Graduate Program, University of Wisconsin School of Medicine and Public Health, Madison WI

Corresponding Author: Rama K Maganti, MD

Address; 1685 Highland Ave, 7142 MFCB

Madison, WI 53705, USA

Email: [maganti@neurology.wisc.edu](mailto:maganti@neurology.wisc.edu)

Phone: 608 265-4352

Fax: 608 263-0412

Figure S1: Sleep scoring in 4 second epochs using Sirenia sleep software is shown. Each panel has a frontal EEG (first tracing), a parietal EEG (second tracing) and an EMG electrode (third tracing). EEG is band pass filtered from 1-70 Hz and EMG was band pass filtered between 5-500Hz prior to scoring sleep. Panel A shows a wake pattern with low amplitude EEG and increased EMG tone. Panel B shows a NREM sleep pattern with high amplitude frontal EEG and sleep spindles as well as a low EMG tone (EKG artifact seen in EMG). Panel C shows rem Sleep pattern low amplitude EEG and low EMG tone.

Figure S2: Data for number of bouts (episodes) of each vigilance state is shown among NC control, Sham injury or TBI group (Mean±SEM is shown). Significance in group differences was indicated by an *. At week 1, ANOVA showed a main effect of group for wake (F (2 21) = 6.095; *p*=0.008); NREM (F (2 21) =9.9706, *p*<0.001) and REM (F (2 21) =9.171, *p*=0.001). At month 2 main effect of group was seen only for wake (F (2 22) =4.08; *p*=0.03). On post-hoc Bonferroni corrections, Sham group had greater number of wake bouts compared to NC control (Sham: 119.14±4.35; NC: 81.00±4.35; p<0.01); greater number of NREM bouts (Sham: 148.17±15.06; NC: 83.17±3.03; *p*=<0.01; TBI: 116.91±6.63 *p*=0.003) and REM bouts (Sham: 89.71±11.27; NC: 40.17±5.33, *p*<0.01). At month 2, TBI group had significantly higher wake bouts compared to NC controls (TBI: 123.17±5.81; NC control: 81.00±4.35; *p*=0.03).

Figure S3: Data of sleep bout length analysis is shown wake (A and D), NREM (B and E) and REM (C and F) in week 1 and month 1 or 2. Mean bout length (in minutes) and 95%CI for each group as well as p value is shown in each panel. There was a main effect of group on the average bout length for wake (F (2 21) =17.082, *p*<0.001) and REM (F (2 21) =9.286, *p*=0.001) but not for NREM (*p*=0.09) at week 1. Similarly, at month 1 or 2 also there was a main effect of group in average bout length of wake (F (2 22) =7.138, *p*=0.004) and NREM (F (2 22) =4.32, *p*=0.02), but not for REM. On post-hoc Bonferroni-Holm corrections, the average wake bout was significantly longer for NC (568.54±25.88) compared to Sham (290.82±36.78, *p*<0.01) or TBI (342.33±29.81, *p*<0.01) at week 1 and at month 1 or 2 (NC:479.01 ± 26.70; Sham: 319.29 ± 42.60, *p*<0.01 and TBI: 339.39 ± 20.79, *p*<0.01). For NREM, post-hoc comparisons showed that at month 1 or 2, NC (498.16±22.20) had longer average bout length than TBI (348.51±22.57, *p*<0.05) but not different from Sham (454.66±66.58, *p*=0.48). For REM at week 1, on post-hoc comparisons, NC (111.12±3.27) has longer average REM bout length compared to Sham (83.10±3.93, *p*<0.01) or TBI (83.29±5.21, *p*<0.01) but no difference was seen between Sham and TBI. (Figure S2).

Figure S4: Ex vivo CT imaging data is shown for **(A, top row)** five brains from animals that had TBI. Notice the lesion produced by TBI on the right hemisphere (left side of image). **(B, bottom row)** five brains from Sham animals that received a craniotomy without TBI. **(C)** differences in volume of lesion at the TBI/craniotomy site is statistically significant. (Sham: 0.82 ± 0.68; TBI: 10.49 ± 7.2 mm3; p = 0.039).

Table S1: Data is shown for time spent awake only across the 24 hours in six 4-hour bins among NC control, Sham and TBI at week 1 and Month 1 or 2. Analysis using repeated measures of ANOVA showed main effect of time and an oscillation pattern both in week 1 [F(5 17)=6.74; p=0.003] and at month 1 or 2 [F(5 17)=6.78; p=0.003], where the time spent in Wake was lower in Bins 1 and 2 (lights on) and gradually increased in Bins 3 and 4) during lights off before declining in the last bin (prior to lights on).

Table S2: Results from post-hoc multiple comparisons tests associated with the ANOVA analyses of NREM delta power graphically represented in Figure 5 are shown here. All values are mean ± 95% confidence interval. Panels A-C summarize the binned analysis for NC control, SHAM and TBI groups at acute and chronic timepoints, showing individual means (panel A), bin values of all groups (panel B), and group means (panel C). Panels D-F summarized the binned analysis of TBI animals, with or without post-traumatic seizures, at acute and chronic timepoints, showing individual means (panel D), mean bin values of both groups (panel E), and group means (panel F).

Table S3: The density of spindles across time in 4 six-hour bins is shown. Note that the spindles increase from Bin 1-3 before declining. Mixed model ANOVA showed the main effect of time (F (2 21) =8.02, *p*=0.001). However, no interaction was seen between rows and columns (p=0.37). In addition, one-way ANOVA showed no difference between the groups in each time bin (data shown in the table).

Table S4: Table shows the different spindle characteristics we analyzed including density duration, frequency, amplitude and power. Differences between the groups was analyzed with ANOVA and post-doc Bonferroni-Holm correction. The p values from ANOVA were shown the table. ON post-hoc corrections, spindle duration was higher in NC compared to sham (p<0.01), and CCI (p<0.05) but not different between sham and CCI (p=0.89) at week 1 and month 1 or 2. Similarly, spindle power was higher in NC control compared to sham (p<0.01) or CCI (p<0.01) but CCI was not different from sham (p=0.63) at week 1 or month 1 or 2. Spindle amplitude was lower in NC compared to sham (p<0.05) or CCI (p<0.05) at week 1 only. No differences were seen between the groups in spindle density or frequency at week 1 or month 1 or 2.

Figure S1:


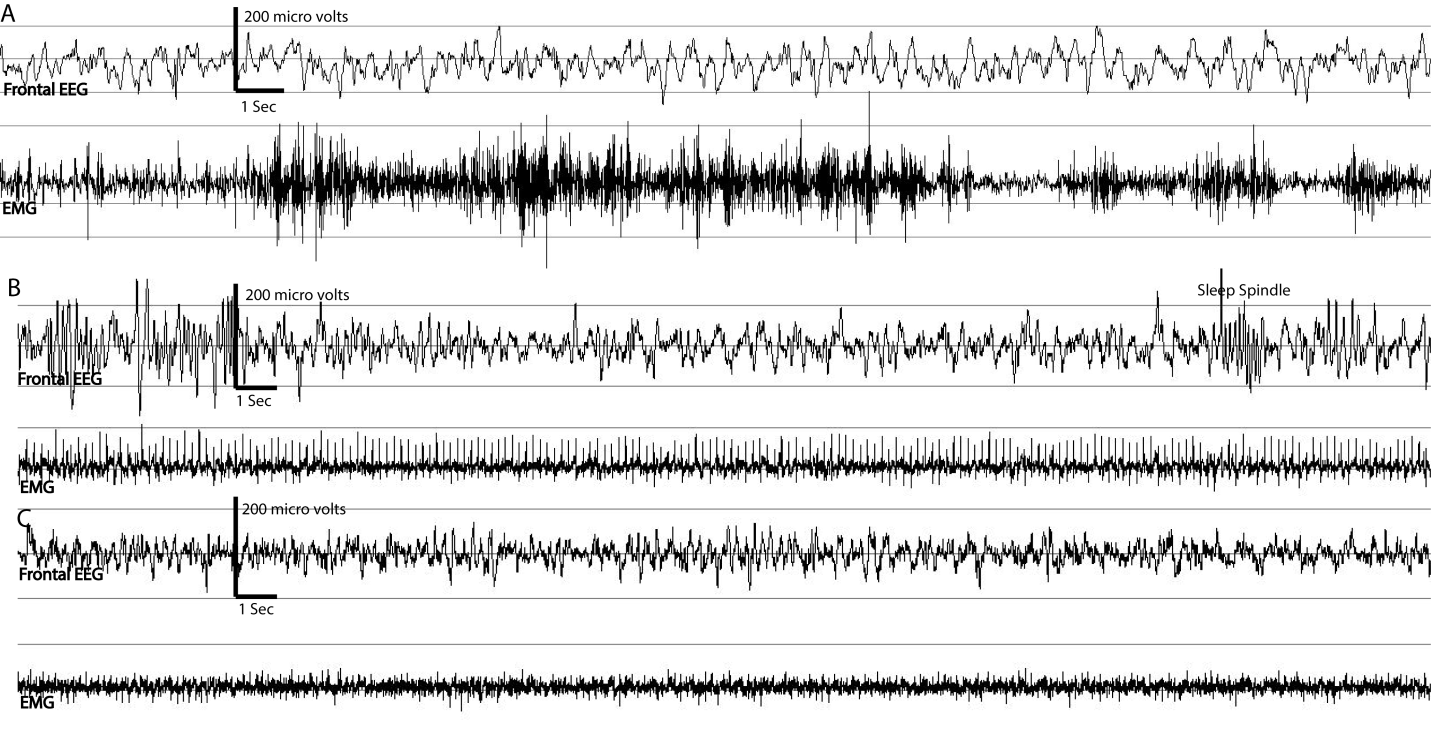


Fig ure S2:


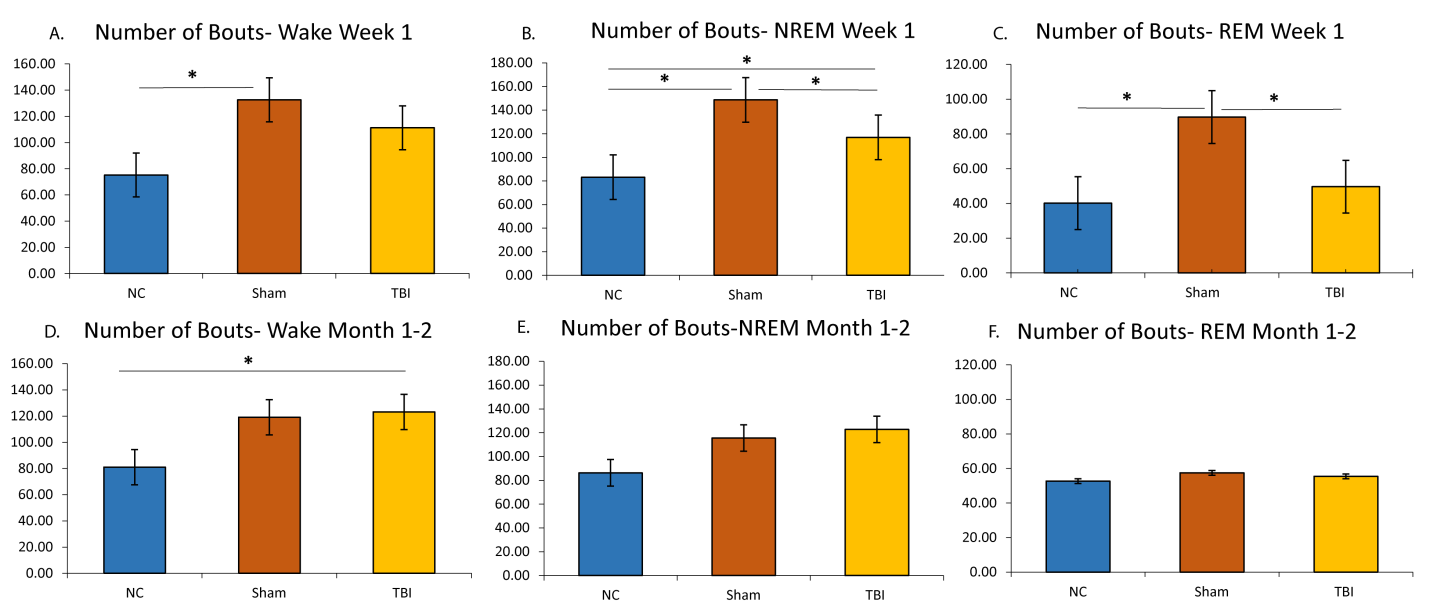


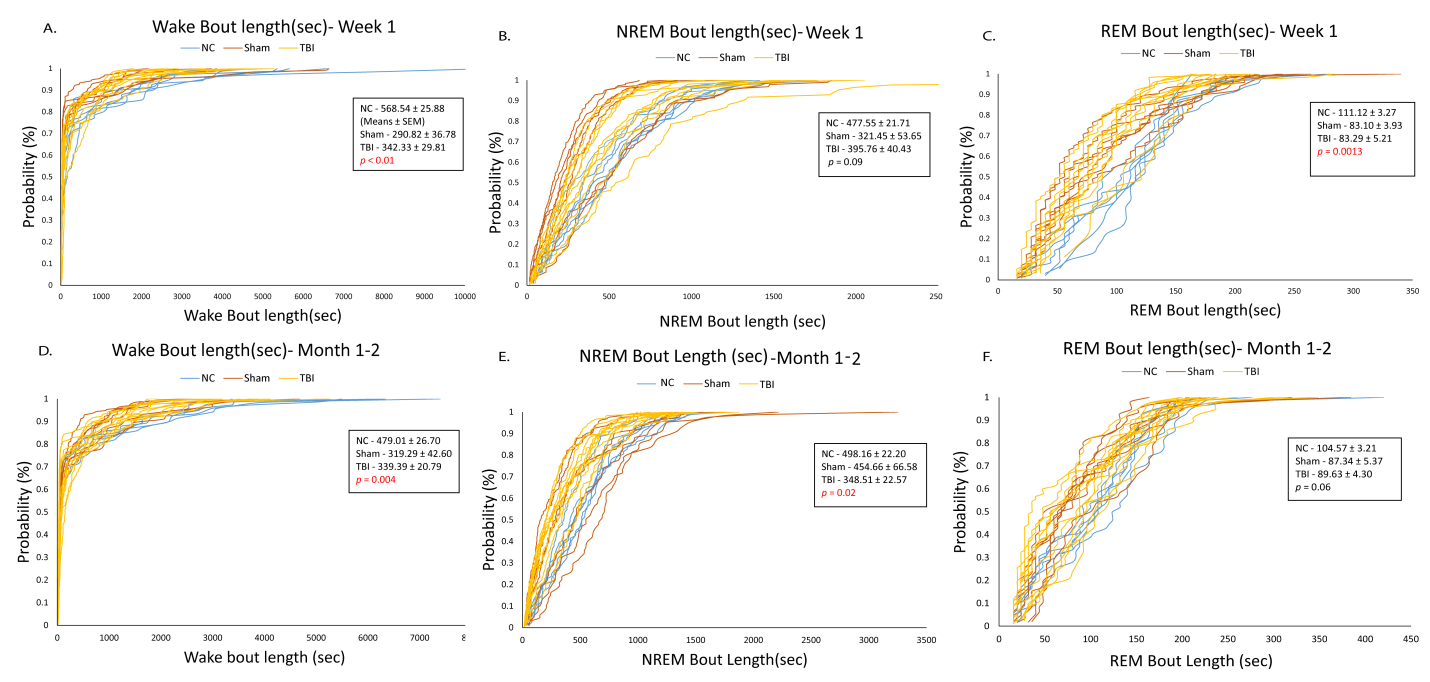
Figure S3

Figure S4:


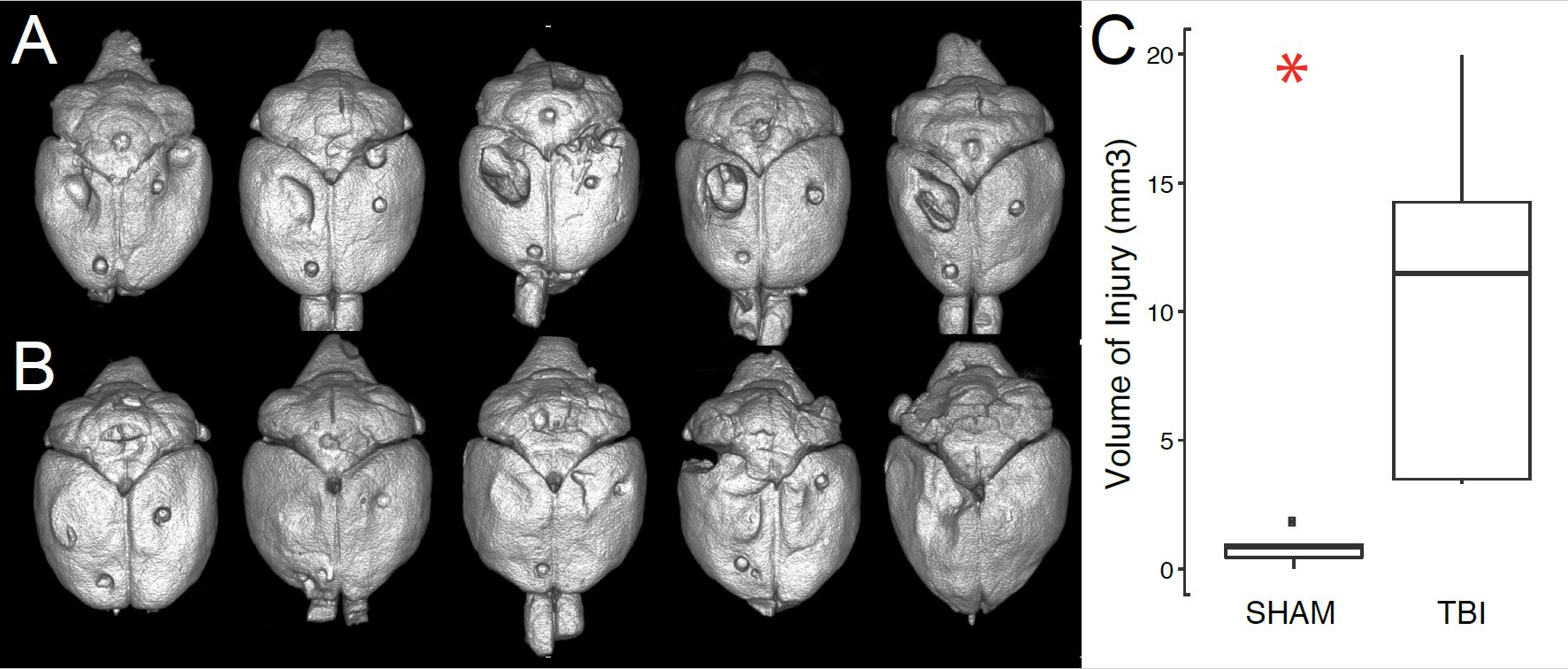


| Table S1: Time spent in Wake state in minutes, in each of the 4-hour bins | | | |
| --- | --- | --- | --- |
| Week 1 of Recording | | | |
| Time Bin | NC control | Sham | CCI |
| Bin 1 | 90.46±5.43 | 89.83±19.06 | 89.25±33.99 |
| Bin 2 | 79.31±3.65 | 77.99±18.22 | 82.88±21.46 |
| Bin 3 | 117.09±6.0 | 111.24±26.7 | 112.5±34.43 |
| Bin 4 | 131.89±5.21 | 124.74±24.2 | 120.02±14.54 |
| Bin 5 | 116.11±7.07 | 105.91±29.6 | 110.37±36.15 |
| Bin 6 | 96.60±7.04 | 79.33±28.73 | 99.16±38.53 |
| Month 1 or 2 of recording | | | |
| Bin 1 | 85.82±8.36 | 73.43±21.06 | 93.18±17.18 |
| Bin 2 | 62.37±19.12 | 67.12±19.72 | 72.77±18.14 |
| Bin 3 | 104.42±13.76 | 104.44±13.76 | 122.46±18.53 |
| Bin 4 | 139.53±10.87 | 117.90±18.21 | 152.68±18.54 |
| Bin 5 | 131.45±18.60 | 103.94±16.80 | 117.55±18.08 |
| Bin 6 | 115.83±27.41 | 98.68±20.82 | 119.15±19.09 |

Table S2:


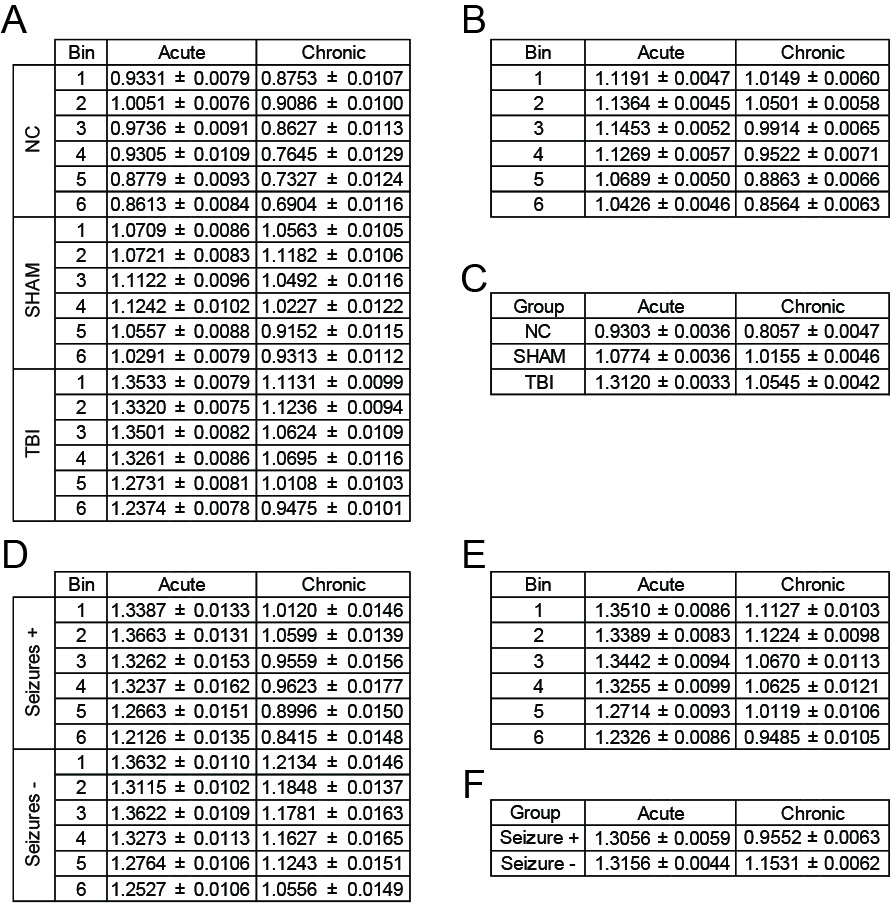


| Table S3. Spindle density shown in 4 six-hour bins among the NC, sham and CCI groups | | | | |
| --- | --- | --- | --- | --- |
|  | NC control | Sham | CCI | One-way AVOVA |
| Time Bin 1 | 2.26±0.52 | 2.90±0.83 | 2.84±0.65 | F(2, 35) = 0.13; p=0.87 |
| Time Bin 2 | 2.88±0.35 | 3.71±0.42 | 4.64+1.44 | F(2, 36) = 1.86; p=0.16 |
| Time Bin 3 | 3.35±0.20 | 6.01±1.61 | 4.46±0.81 | F(2, 36) =2.13; p=0.13 |
| Time Bin 4 | 2.28±0.29 | 2.73±0.25 | 2.85±0.96 | F(2, 36)=0.54; p=0.58 |

| Table S4: Spindle characteristics in different groups, analyzed with ANOVA | | | | | |
| --- | --- | --- | --- | --- | --- |
| Week 1 | Density (number per min) Mean±SD | Duration (in seconds) Mean±SD | Frequency (in Hz) Mean±SD | Amplitude (in µV) Mean±SD | Power (µV^2^ Mean±SD) |
| NC Control | 2.74±0.48 | 2.14±0.28 | 12.09±0.30 | 205.33±34.74 | 668.99±239.22 |
| Sham Injury | 2.89±0.70 | 1.83±0.21 | 11.83±0.37 | 397.62±215.09 | 339.21±131.31 |
| TBI | 3.21±0.56 | 1.78±0.31 | 11.77±0.39 | 397.62±215.09 | 379.26±115.65 |
| F(2, 30) | 2.104 | 5.58 | 2.53 | 5.30 | 10.95 |
| P value | 0.14 | 0.009 | 0.09 | <0.001 | <0.001 |
| Month 1 or 2 | | | | | |
| NC Control | 2.63±062 | 2.22±0.27 | 12.39±0.39 | 226.41±36.36 | 996.92±246.04 |
| Sham | 3.21±035 | 1.64±0.17 | 11.88±0.51 | 203.47±62.22 | 442.78±259.37 |
| TBI | 2.91±0.37 | 1.92±0.23 | 12.16±0.39 | 392.48±212.64 | 586.52±265.01 |
| F(2, 19) | 2.141 | 8.36 | 1.90 | 3.44 | 6.52 |
| P value | 0.14 | 0.003 | 0.17 | 0.055 | 0.007 |

Table S4:
